# *Lactobacillus fermentum* PS150 promotes non-rapid eye movement sleep in the first night effect of mice

**DOI:** 10.1038/s41598-021-95659-3

**Published:** 2021-08-11

**Authors:** Alexander Lin, Ching-Ting Shih, Hsu-Feng Chu, Chieh-Wen Chen, Yu-Ting Cheng, Chien-Chen Wu, Cheryl C. H. Yang, Ying-Chieh Tsai

**Affiliations:** 1 Institute of Biochemistry and Molecular Biology, National Yang-Ming University, 155, Section 2, Linong Street, Beitou District, Taipei, 11221 Taiwan; 2 Chung Mei Biopharma Co., Ltd., Taichung, Taiwan; 3 Biomedical Industry Ph.D. Program, National Yang-Ming University, Taipei, Taiwan; 4 Institute of Brain Science, National Yang-Ming University, No. 155, Section 2, Linong Street, Beitou District, Taipei, 11221 Taiwan; 5 Sleep Research Center, National Yang-Ming University, Taipei, Taiwan; 6 Bened Biomedical Co., Ltd., Taipei, Taiwan; 7 Brain Research Center, National Yang-Ming University, Taipei, Taiwan

**Keywords:** Circadian rhythms and sleep, Microbial communities, Microbiology, Neuroscience

## Abstract

The first night effect (FNE) is a type of sleep disturbance caused by an unfamiliar environment, which leads to difficulty falling asleep and reduced sleep duration. Previously, we reported that *Lactobacillus fermentum* PS150 (PS150) improves sleep conditions in a pentobarbital-induced sleep mouse model. In this study, we aimed to evaluate the effect of PS150 on the FNE in mice. Briefly, mice were implanted with electrodes and orally administered PS150 for four weeks, and then the FNE was induced by cage changing. Analysis of polysomnographic signals revealed that intervention with PS150 restored non-rapid eye movement (NREM) sleep length under the FNE. Compared to diphenhydramine, a commonly used sleep aid, PS150 had no unwanted side effects, such as rapid eye movement (REM) sleep deprivation and fragmented sleep. Moreover, temporal analysis revealed that PS150 efficiently reduced both sleep latency and time spent restoring normal levels of REM sleep. Taken together, these results suggest that PS150 efficiently ameliorates sleep disturbance caused by the FNE. Additionally, V3–V4 16S rRNA sequencing revealed significant increases in Erysipelotrichia, Actinobacteria, and Coriobacteriia in fecal specimens of the PS150-treated group, indicating that PS150 induces gut microbiota remodeling.

## Introduction

Insomnia, which comprises difficulties in maintaining or initiating sleep, is the most common sleep disorder in the general population. Greater than 20% of adults suffer from chronic insomnia^1^. Many factors that cause chronic insomnia, such as shift work, irregular work hours, jet lag, and stress, can be linked to modern lifestyles^2^. In addition to chronic factors, sudden changes in the environment, such as traveling or moving to a new place, also cause insomnia. This is called the first night effect (FNE), which can lead to various symptoms, including reduced total and rapid eye movement (REM) sleep length, reduced sleep efficiency, fragmented sleep, and increased sleep latency^3,4^. Therefore, FNE is considered a general sleep disturbance that potentially affects quality of life.

Insufficient sleep can lead to memory loss, irritability, depression, distraction, and fatigue. In addition to cognitive functions, sleep disorders are also correlated with metabolic syndromes, such as obesity, inflammation, diabetes, and cardiovascular diseases^5,6^. Although many drugs are readily available to treat insomnia, including benzodiazepine receptor agonists, antihistamines, melatonin receptor agonists, anxiolytics, antidepressants, and antipsychotics, the potential issue of drug dependence and abuse is concerning^7^. Moreover, these drugs are often accompanied by side effects, such as dizziness, headache, drowsiness, amnesia, and cognitive impairment, even leading to an increased risk of mortality^1,8^. Therefore, it is important to identify a potential hypnotic auxiliary to replace—or reduce the use of—these hypnotic drugs with safer options that improve sleep quality and efficiency without significant adverse effects.

Proposed by the FAO/WHO in 2002, probiotics are defined as living microorganisms that promote the health of the host^9^. Increasing evidence shows that long-term intake of probiotics improve health with no apparent side effects^10–13^. Therefore, numerous potential functions of probiotics have been proposed, even in the field of mental health. Possibly mediated by the immunoregulatory, neuroendocrine, or vagus nerve pathways, the microbiome-gut-brain axis (MGBA) explicitly refers to bidirectional signaling communication between the gastrointestinal and central nervous systems through the intestinal microbiome^14–16^. Probiotics that confer mental or behavioral benefits through the MGBA are called psychobiotics and have promising potential for future applications^17^.

Studies have shown that sleep disturbance leads to metabolic consequences accompanied by mild gut microbiota alterations^18–20^. Recently, some studies have reported preliminary evidence that the gut microbiota affects sleep conditions. Chronic antibiotic-treated mice exhibit disrupted sleep architecture accompanied by intestinal neurotransmitter alterations^21^. In addition, dietary prebiotics were found to effectively improve sleep conditions affected by stress^22^. Some novel microbial-dependent fecal metabolites have been proposed to improve sleep^23^. These results suggest the possible application of remodeling the microbiota to improve sleep quality of the host through the MGBA.

Numerous *Lactobacillus* spp. and *Bifidobacterium* spp*.* have been identified as effective psychobiotics against depression, anxiety, and even neurodegenerative disorders ^24^. Our previous studies demonstrated that oral administration of *Lactobacillus fermentum* PS150 (PS150) inhibits inflammation, reduces hypothalamic–pituitary–adrenal (HPA) axis activation, increases sleep duration, decreases sleep latency, and prevents caffeine-induced sleeplessness in experimental rodents^25,26^. These results suggest the potential application of PS150 as a daily supplement to alleviate sleep disturbance. Apart from the unstressed pentobarbital-induced sleep model in our previous study, we aimed to evaluate the effect of PS150 in a spontaneous sleep model with mild stress, which is more biologically representative. In this study, we applied electroencephalogram (EEG) and electromyogram (EMG) recordings to evaluate the effect of PS150 intervention on spontaneous sleep. Furthermore, since studies have shown a possible connection between the microbiota and sleep, 16S rRNA sequencing was performed to explore the possible effect of PS150 on the gut microbiota.

## Results

### PS150 prevents insomnia caused by FNE

**Figure 1:**
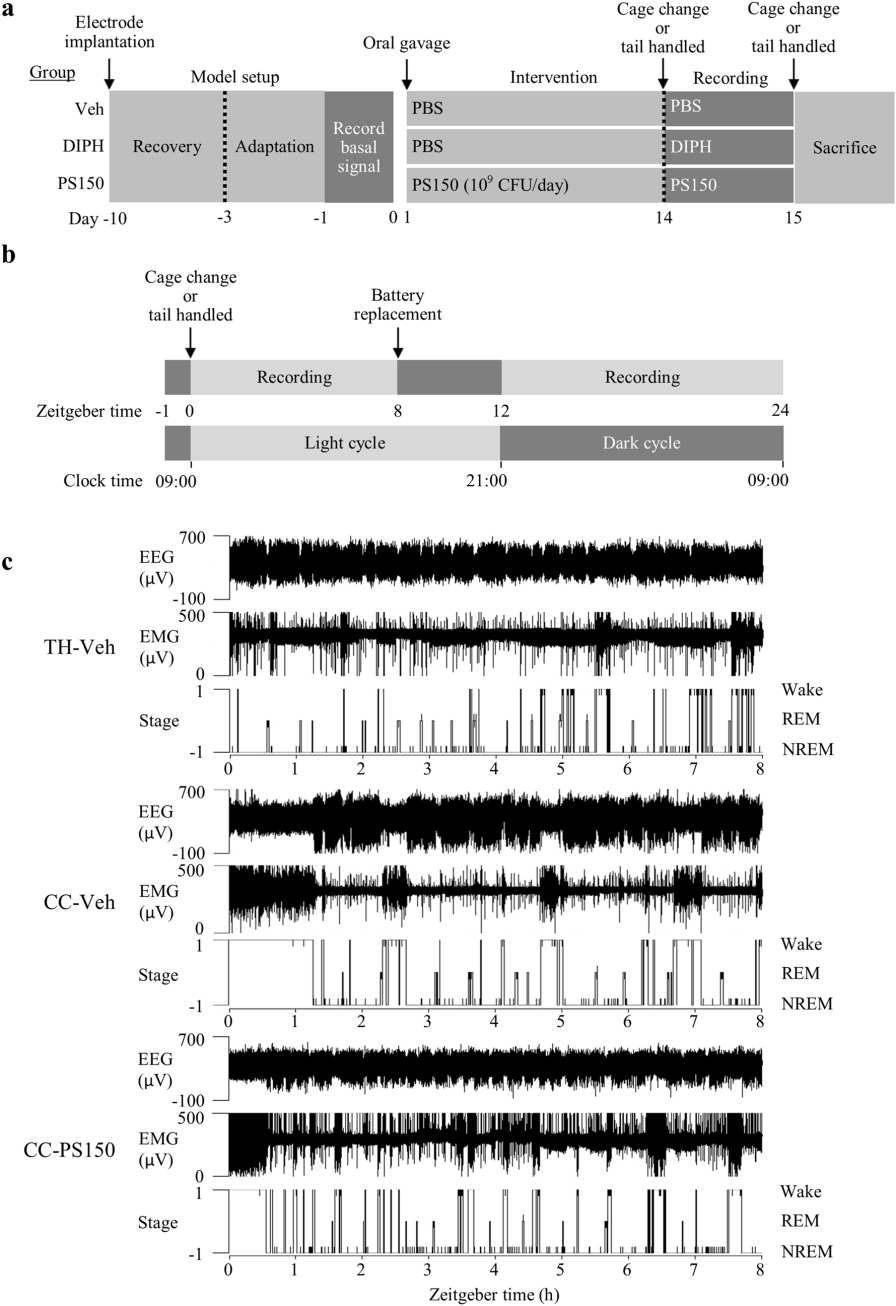
Schematic view of the experimental design for polysomnographic recording. **(a)** Experimental flow chart of the animal model. The probiotic intervention was performed 10 days after electrode implantation and proceeded for 14 days. The cage change procedure was performed before polysomnographic recording on day 14 and repeated before sacrifice on day 15. **(b)** Experimental flow chart of polysomnographic recording on day 14. Zeitgeber time (ZT) 0 was defined as 0900 h when the light was turned on. **(c)** Representative polysomnographs. Continuous analysis of representative data recorded at ZT 0–8 from tail-handled control (TH-Veh), cage change control (CC-Veh), and cage change PS150-treated (CC-PS150) groups are shown.

**Figure 2:**
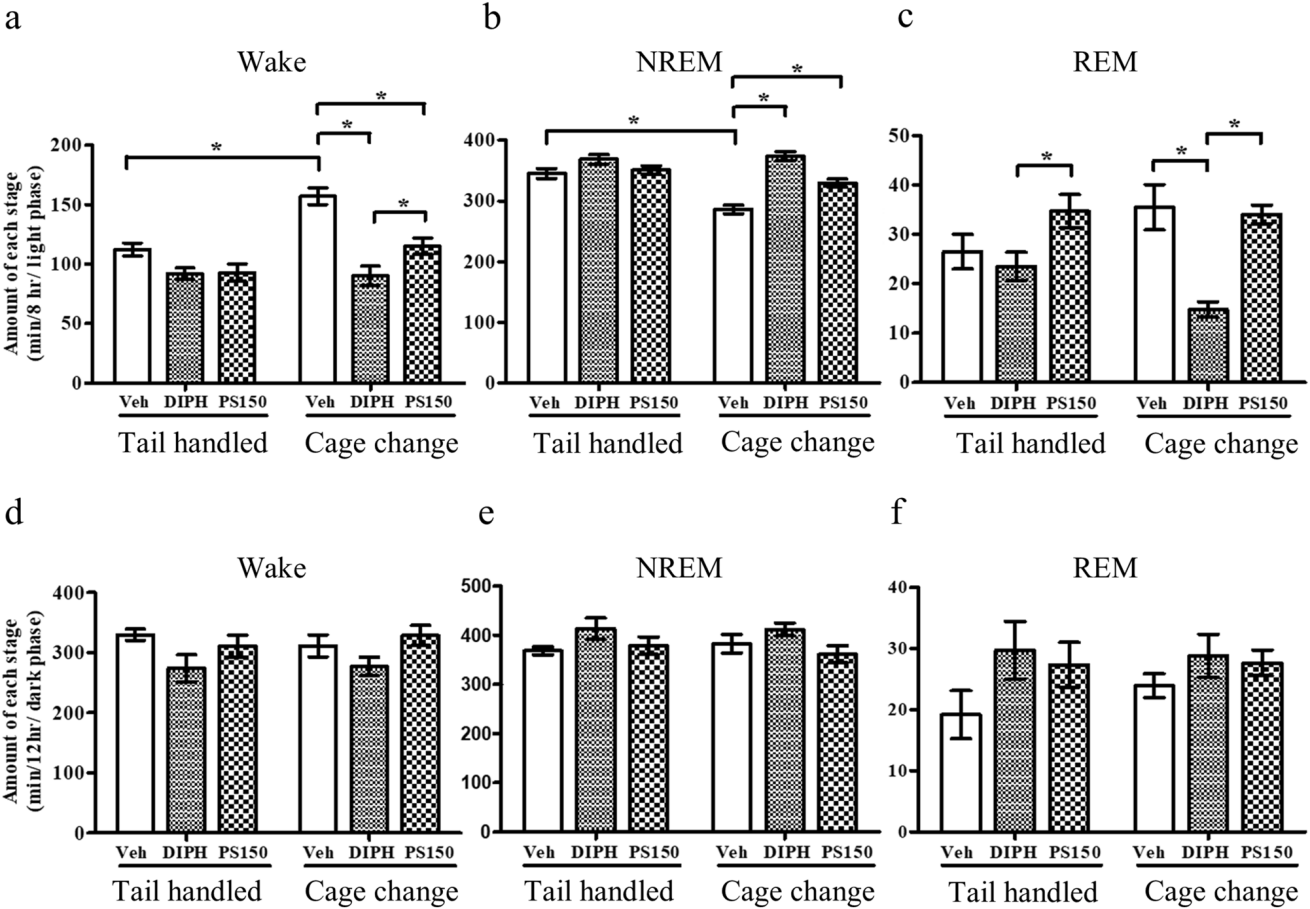
Total time spent in vigilance and sleep states during the light or dark phase. **(a–c)** The total amount of wake, non-rapid eye movement (NREM), and REM states in the 8-h recording period of the light phase (ZT 0–ZT 8). **(d–f)** The total amount of wake, NREM, and REM states in the 12 h recording period of the dark phase (ZT 12–ZT 24). Data are expressed as the mean ± SEM (*n* = 8–9) and were analyzed by two-way ANOVA with Bonferroni correction (*p < 0.05).

To minimize exterior alteration, a wireless recording system and a noninvasive cage change model were applied to evaluate the effect of PS150 on FNE (Fig. 1a). Briefly, mice were orally administered vehicle (phosphate-buffered saline), diphenhydramine (DIPH), or PS150 immediately before the cage change procedure. Subsequently, mice in each group were divided into cage change (CC-Veh, CC-DIPH, and CC-PS150) and tail-handled (TH-Veh, TH-DIPH, and TH-PS150) groups. The cage change procedure and polysomnographic recordings were performed at Zeitgeber time (ZT) 0 to record the vigilance stages of mice (Fig. 1b). Two periods (ZT 0–8 and ZT 12–24) were recorded and fast Fourier transformed for further analysis (Fig. 1c). To provide an overview of the sleep condition, we calculated the total length of all three vigilance states of sleep during the two recording periods. The first recording period (ZT 0–8) lies in the light phase, which is the inactive time for mice in their normal circadian rhythm. The cage change group exhibited significantly increased wakefulness and reduced non-rapid eye movement (NREM) sleep, suggesting a successful mimic of the FNE in this animal model (Fig. 2a,b). Intervention using DIPH, an antihistamine used to treat insomnia, efficiently ameliorated the effect of FNE. More importantly, the PS150 group exhibited prolonged NREM sleep in response to cage change stress (Fig. 2b). Interestingly, DIPH treatment significantly reduced REM sleep, which was not affected in the PS150 group (Fig. 2c). The distribution of wake and sleep bouts was also affected by FNE. The number of wake bouts longer than 300 s was significantly increased in the CC-Veh group. However, this effect was efficiently ameliorated by DIPH and PS150 (Fig. S1a). Additionally, we discovered that treatment with either DIPH or PS150 led to an increase in the number of shorter sleep bouts (Fig. S1b). These results suggest that administration of PS150 alleviates the effects of FNE. We further analyzed the second recording period (ZT 12–24), which lies mostly in the dark phase. None of the states were significantly different between any of the groups in the dark phase (Fig. 2d–f).

### PS150 and DIPH modify sleep architecture in different manners

**Figure 3:**
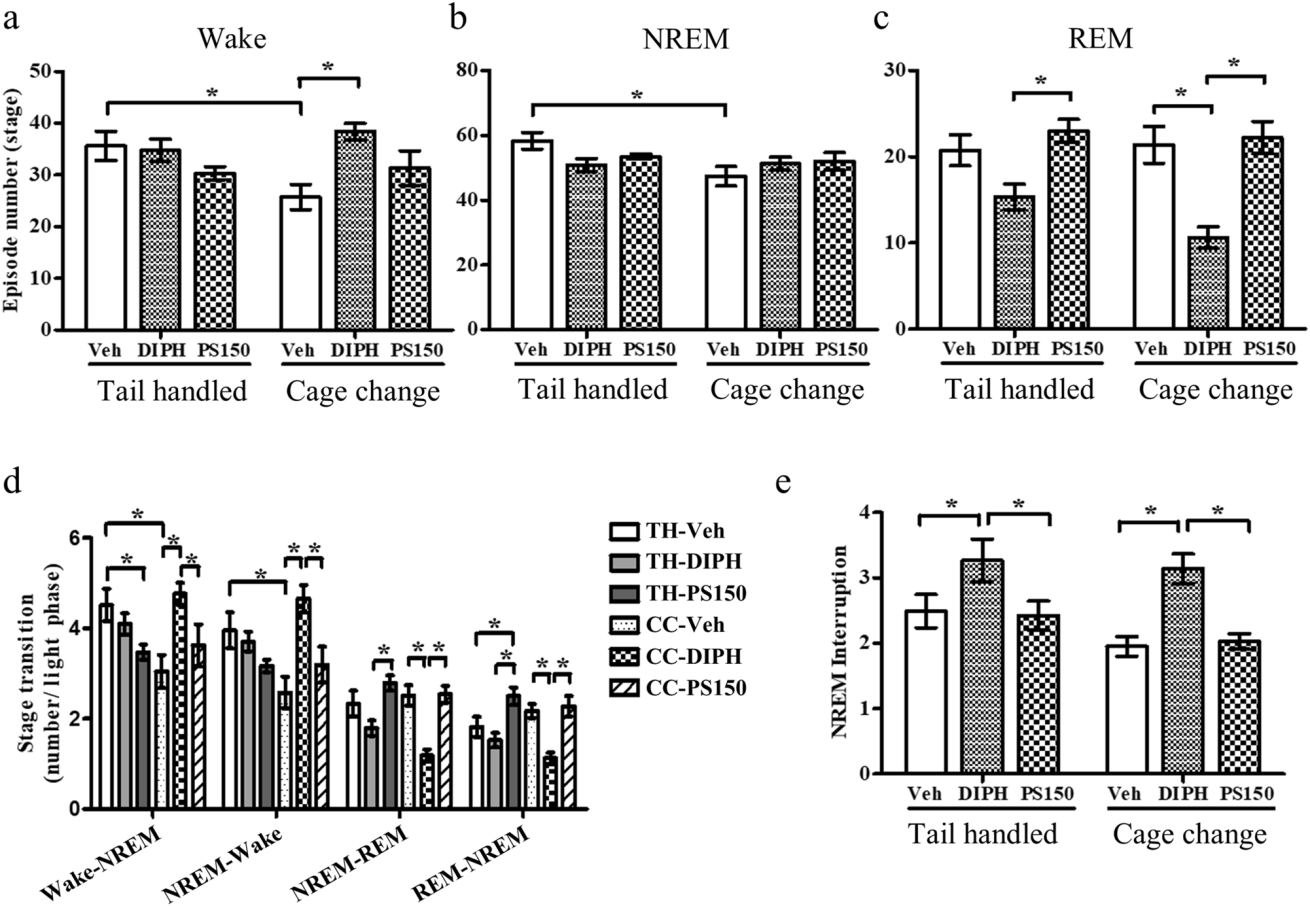
Total number of vigilance and sleep states during the light phase. **(a–c)** The total number of wakefulness, non-rapid eye movement (NREM), and REM states. **(d)** The average number of transitions per hour between each state. **(e)** The total number of NREM sleep interruptions observed. Data are expressed as the mean ± SEM (*n* = 7–9) and were analyzed by two-way ANOVA with Bonferroni correction (*p < 0.05).

To explore the differences in sleep architecture between oral administration of DIPH and PS150 in the cage change model, we evaluated the number of episodes, interruption times, and state transitions during the light phase. The cage change procedure significantly reduced the number of wakefulness and NREM sleep events (Fig. 3a,b). This effect was partially reversed by the DIPH intervention, which significantly increased the amount of wakefulness (Fig. 3a). Furthermore, the number of REM sleep episodes was affected by DIPH in which the cage change (CC-DIPH) group exhibited a significant decrease compared to the control group (CC-Veh) under FNE (Fig. 3c). In contrast, no significant alteration in episode number was caused by PS150 compared to the vehicle group. The reduced wakefulness (Fig. 2a) and increased number of wake stages (Fig. 3a) indicate shorter average wakefulness caused by DIPH in the cage change group, indicating that DIPH and PS150 may modulate sleep architecture by distinct mechanisms, not only with respect to the length of NREM and REM sleep but also the transition between vigilance and sleep stages.

### PS150 retains a normal stage transition under FNE

To clarify the differential effects of PS150 on vigilance and sleep state transition, we analyzed the state transition number between each state. The cage change procedure led to a significant decrease in the number of transitions between wakefulness and NREM sleep. However, it did not affect the NREM–REM transition. In contrast, the NREM–REM transition in the DIPH group was significantly reduced, which was not observed in the PS150 group (Fig. 3d). The reduced total length, number of transitions, and number of REM sleep episodes (Figs. 2c, and 3c) suggest that DIPH leads to REM sleep deprivation; however, PS150 did not affect REM sleep status. For the DIPH group under FNE, we observed a significantly increased frequency of wake–NREM transitions, consistent with our previous observation (Fig. 3d). Moreover, the frequency of NREM interruption in response to FNE was significantly increased by DIPH intervention, suggesting worse sleep quality (Fig. 3e). Collectively, these results demonstrate that PS150 does not affect sleep architecture. In contrast, DIPH treatment induces REM sleep deprivation and NREM sleep fragmentation, ultimately leading to unstable sleep architecture.

### Oral administration of PS150 reduces sleep latency

**Figure 4:**
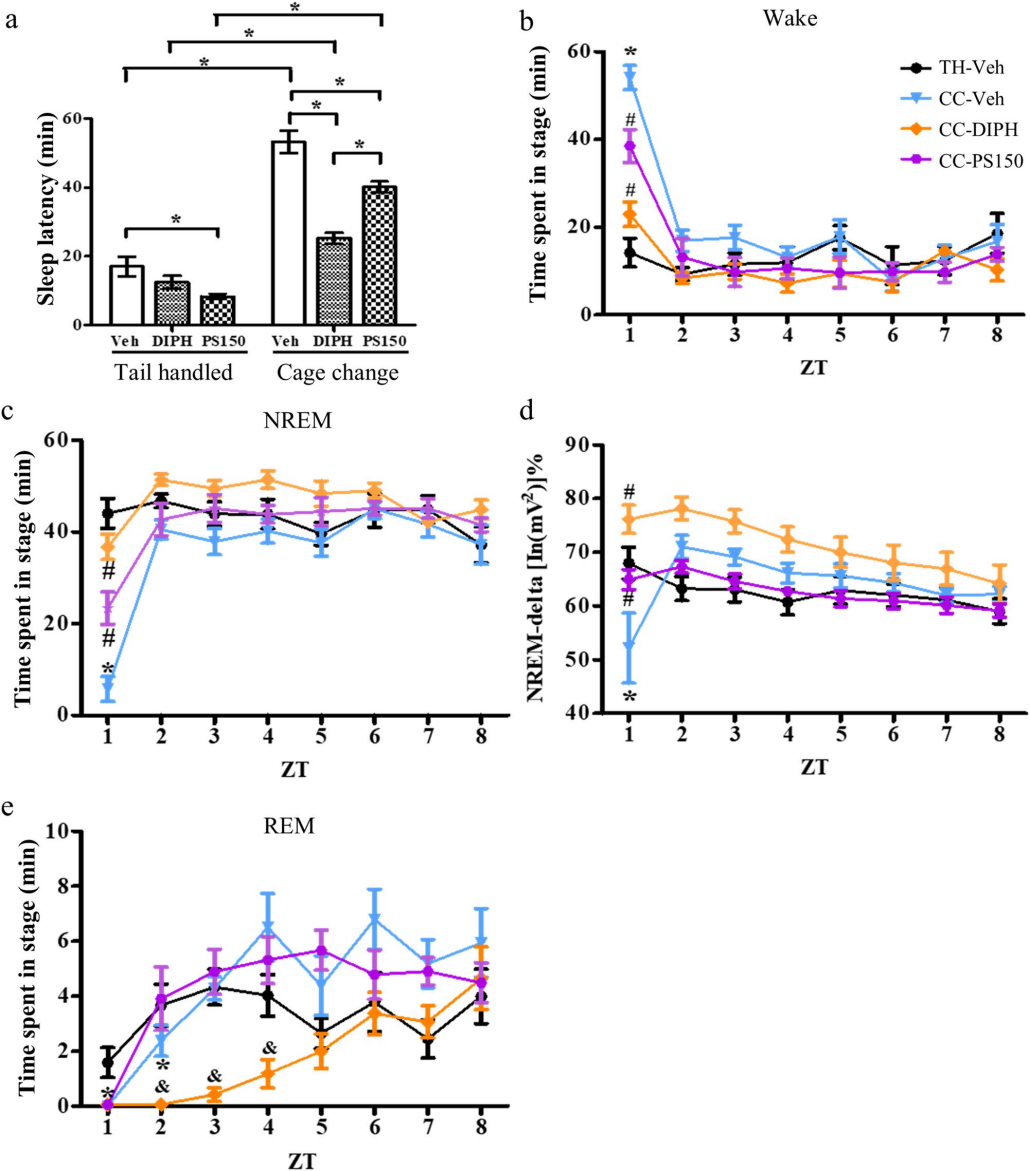
Time-dependent alterations in vigilance and sleep stages during the light phase. **(a)** Sleep latency of each group. Latency was defined as the time between the cage change procedure and the first non-rapid eye movement (NREM) sleep that lasted longer than 5 min. **(b,c)** The amount of wake and NREM states in each hour. Data from the unchanged control (TH-Veh), cage change control (CC-Veh), cage change positive control (CC-DIPH), and cage change PS150-treated (CC-PS150) groups are colored black, blue, orange, and violet, respectively. **(d)** The relative intensity of NREM-delta power in each hour. **(e)** Accumulated amount of REM states in each hour. Data are expressed as the mean ± SEM (*n* = 7–9). For sleep latency, data were analyzed using two-way ANOVA with Bonferroni correction. For the effect of treatment in each hour, data were analyzed using one-way ANOVA with Tukey’s post hoc test. (*p < 0.05, compared to TH-Veh; ^#^*p* < 0.05, compared to CC-Veh. ^&^*p* < 0.05, compared to CC-Veh and CC-PS150).

Previously, we reported significantly reduced sleep latency in response to PS150 treatment in a pentobarbital-induced sleep mouse model. Similar to our previous discovery, we observed that PS150 intervention significantly reduced sleep latency in both the cage change and tail-handled groups. (Fig. 4a). Therefore, we inferred that PS150 may enhance sleep initiation. Subsequently, we plotted the accumulated time of vigilance and sleep stages at each hour to verify the temporal alteration caused by PS150. The results showed that mice under FNE stayed almost fully awake during the first hour (Fig. 4b), whereas both PS150 and DIPH intervention ameliorated this effect. A similar phenomenon was also observed in NREM sleep, where both PS150 and DIPH intervention significantly increased the length of NREM sleep during the first hour (Fig. 4c). Notably, as FNE leads to a major decrease in NREM-delta power, DIPH and PS150 treatment restored the amount of delta power to a normal level (Fig. 4d). Complete loss of REM sleep during the first hour was observed in all cage change groups. Intervention with PS150 revealed a more rapid recovery of REM sleep than that in the vehicle control. REM sleep in the control group returned to normal levels in the third hour, whereas the PS150-treated group returned to normal levels in the second hour. In contrast, the reduction in REM sleep persisted to the fourth hour in the DIPH-treated group (Fig. 4e).

### PS150 may improve sleep conditions through a noncanonical pathway

Our results clearly demonstrated the beneficial effect of PS150 on FNE in a spontaneous sleep model. Next, we aimed to improve our understanding of the underlying mechanism of PS150. Psychobiotics have been reported to decrease proinflammatory cytokines and suppress HPA activity^17^. Therefore, we determined levels of corticosterone in the serum to determine whether PS150 promotes sleep efficiency by affecting HPA axis activity. The results revealed that PS150 reduced corticosterone levels in the tail-handled group. However, no significant difference was found in the cage change group, suggesting that PS150 is unlikely to improve sleep conditions by suppressing the HPA axis (Fig. S2). Since PS150 treatment prolongs NREM sleep, increases NREM-delta power, and shortens sleep latency, we focused on the adenosine and histamine pathways, as they have been reported to be related to NREM sleep and wakefulness. Transcription levels of adenosine A_1_ receptor (A_1_R), adenosine A_2A_ receptor (A_2A_R), ecto-5′-nucleotidase (NT5e), and histamine_1_ receptor (Hist_1_R) were analyzed by quantitative PCR (Table S1). Oral administration of PS150 significantly increased mRNA expression of A_1_R in the hypothalamus rather than the basal forebrain in the tail-handled group. However, this trend was not observed in the cage change group. No significant alteration was found for Hist_1_R.

### PS150 alters fecal microbiota composition in the cage change group

**Figure 5:**
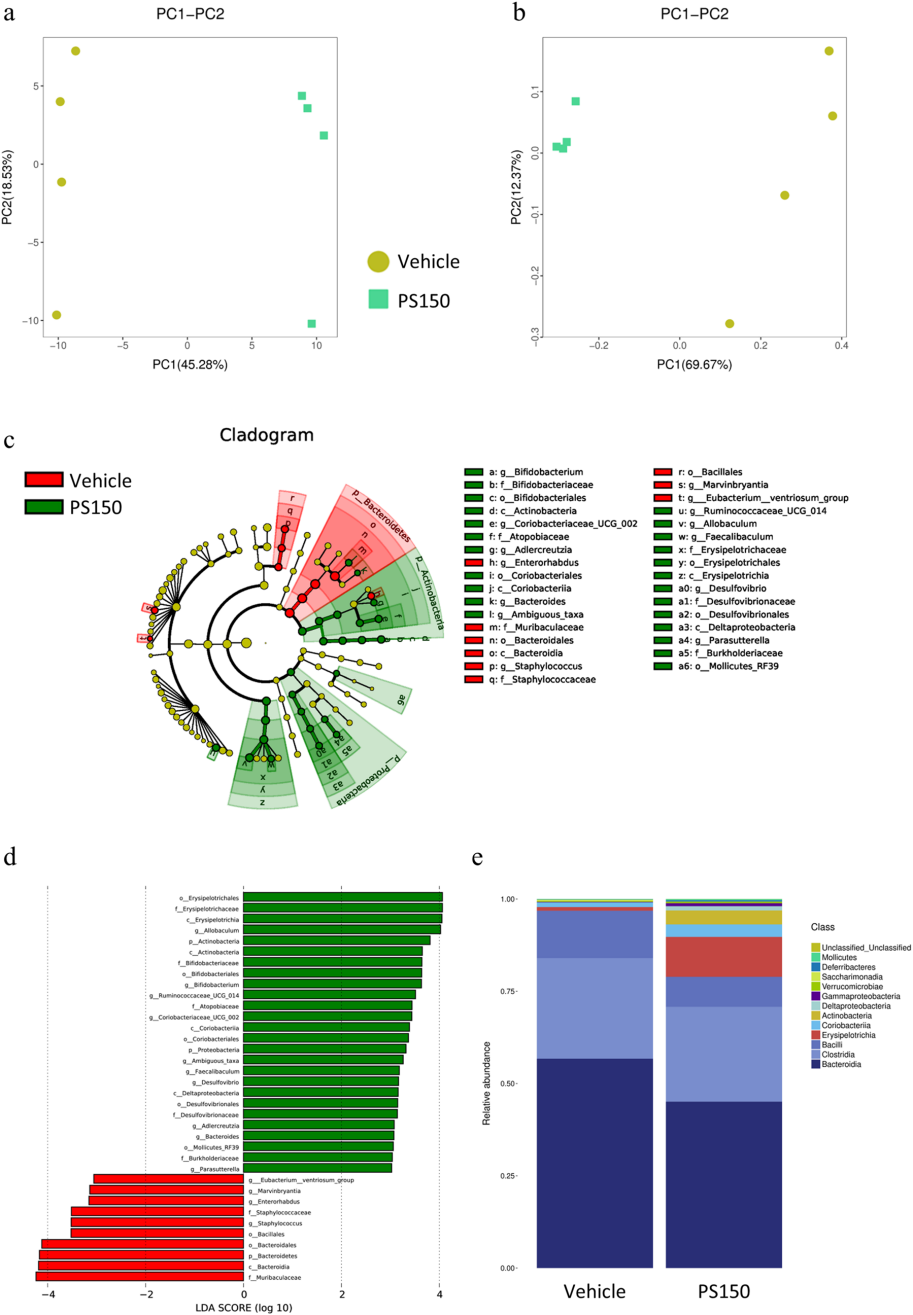
Differential microbiota composition of the fecal specimen from the cage change group. **(a)** PCA and **(b)** PCoA plot of fecal microbiota. **(c)** Cladogram from linear discriminant analysis effect size (LEfSE) and enriched taxonomy in either the control or PS150 group are colored red and green, respectively. **(d) **LDA score from LEfSE analysis. **(e)** The stacked plot of relative microbial distribution at the order level.

To explore possible roles of the gut microbiota in the hypnotic effect of PS150, we applied 16S rRNA sequencing to verify the effect of PS150 in the cage change group. Sequencing was performed with good quality (Table S2) and sufficient read depth (Figure S3a). The results showed that PS150 treatment significantly elevated the Chao1 index, suggesting a major increase in observed species diversity (Table 1). Furthermore, both principal component analysis (PCA) (Fig. 5a) and principal coordinate analysis (PCoA) (Fig. 5b) showed distinct clustering of fecal microbiota composition from either the PS150-administered or control group. The change in beta diversity was further confirmed using both Adonis (*p* = 0.035) and ANOSIM (*p* = 0.037) analysis. In addition to beta diversity, we performed linear discriminant analysis effect size (LEfSE) to identify differentially presented taxa. At the class level, we observed that the abundance of Bacteroidia was decreased in the PS150-treated group. In contrast, Erysipelotrichia, Coriobacteriia, and Actinobacteria were significantly increased in the PS150-treated group (Fig. 5c,d). Notably, the three enriched taxa in the PS150-treated group accounted for approximately 15% of the total microbiota (Fig. 5e), suggesting that PS150 intervention created a competitive advantage for their growth in the gut environment.

**Table 1:** Alpha diversity indexes.

Model	Groups	Chao1		Shannon		Source
		Mean	P-value	Mean	P-value	
The first-night effect	PS150	222 ± 6.1	< 0.001	5.4 ± 0.3	0.624	This study
	Veh	190 ± 5.5		5.2 ± 0.6		
Pentobarbital induced sleep	PS150	288 ± 8.6	< 0.001	5.5 ± 0.4	0.03	^27^
	Veh	237 ± 8.2		5.8 ± 0.2		

Statistical significance was calculated using the *t*-test.

### PS150 alters fecal microbiota composition in naïve mice

Although we observed a significant alteration in the fecal microbiota, this result was hampered by the small sample size (n = 4 in each group). To eliminate this concern, we reanalyzed the fecal samples obtained in our previous study ^25^. Briefly, 19 naïve mice were treated with either vehicle (n = 8) or PS150 (n = 11) for 4 weeks, and their feces were collected for subsequent 16S rRNA sequencing. Overall, all samples were sequenced with sufficient quality (Table S2) and read depth (Fig. S3b). In contrast to our observation under a cage change background, the naïve mice exhibited a significant increase in alpha diversity in both the major and observed species diversity (Table 1). In line with our expectation, both PCA (Fig. S4a) and PCoA (Fig. S4b) analysis revealed distinct differences in microbiota composition. Moreover, both Adonis (*p* = 0.005) and ANOSIM (*p* = 0.001) analyses were statistically significant. We further confirmed the alteration in microbiota using LEfSE analysis (Fig. S4c and S4d). Several taxa in the fecal microbiota were enriched in response to PS150 administration. Similar to previous observations, we identified a significant increase in Erysipelotrichia, Actinobacteria, and Coriobacteriia. The three increased taxa accounted for 19% of the total microbiota (Fig. S4e). In contrast to the aforementioned results, the relative abundance of Bacterioidia was increased in PS150-treated naïve mice. A significant decrease in Clostridia was also observed, which was not affected by PS150 treatment under a cage change background. The occupancy of Erysipelotrichia was highest among the three enriched taxa, and the relative abundance in PS150-treated mice increased by approximately tenfold compared to that in the control group (1.4% to 13.8%). In contrast, enrichment of Actinobacteria was the most dramatic, increasing by more than 40-fold (0.05% to 2.3%). We also observed a threefold increase in Coriobacteriia in the PS150-treated group (1% to 3.2%). Taken together, these results confirm that PS150 intervention induced similar microbiota alterations in two independent animal models.

### PS150 selectively induces microbiota enrichment

Because we observed similar microbiota alterations in response to PS150 administration in both studies, we aimed to inspect altered microorganisms in depth through a comparative study of data from two models. We applied Metastats analysis to explore the differentially presented taxa down to the genus level (Table S3). For data from the naïve mouse model, we applied an FDR cutoff of 0.05 for positive discovery, whereas a *p*-value cutoff of 0.05 was applied for data from this study. A total of 9 and 10 genera were significantly enriched in our previous study and this study, respectively. The genera *Allobaculum*, *Coriobacteriaceae* UCG-002, and *Bifidobacterium*, which belong to Erysipelotrichia, Coriobacteriia, and Actinobacteria, respectively, were consistently elevated in both studies. Notably, the three genera were the dominant taxa in the altered microbiota, which occupied 69% ± 25% of Erysipelotrichia, 70% ± 8% of Coriobacteriia, and 99% ± 1% of Actinobacteria in the PS150-treated groups in both studies. Collectively, our work demonstrated that PS150 administration selectively enriches microbial growth in the host fecal microbiota.

### The remodeled microbiota exhibits distinctive metabolic capability

**Figure 6:**
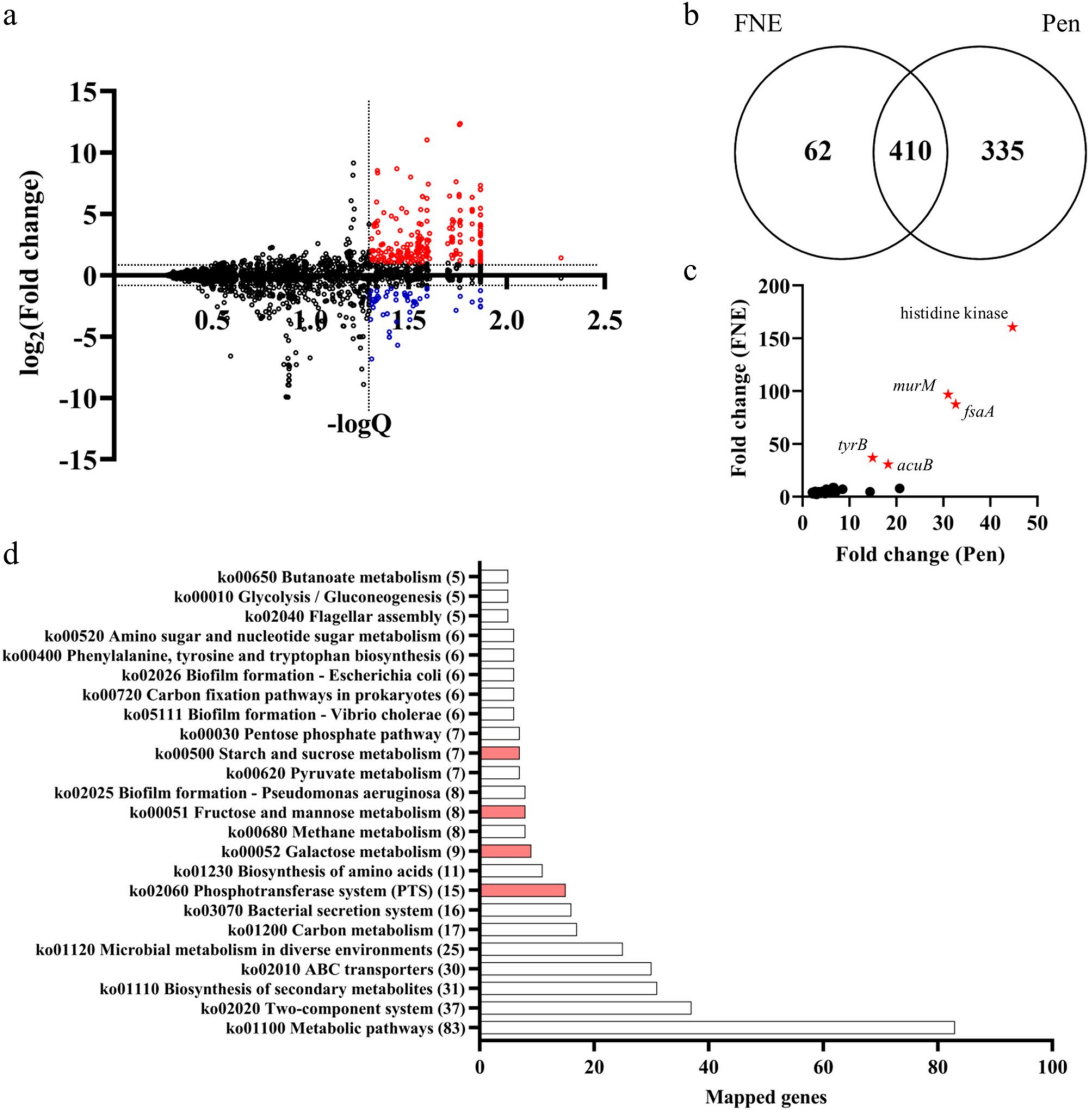
Differential predictive metagenome composition of the fecal microbiota. **(a)** Overall distribution of the predictive metagenome composition. The fold change (PS150/control) of predicted gene copy numbers from the cage change group was plotted against statistical significance (Q value). Significantly enriched and reduced genes in the PS150 group are colored red and blue, respectively. **(b)** Venn diagram of significantly enriched genes from this study (FNE) and our previous pentobarbital-induced sleep model (Pen). **(c)** Abundant genes enriched in the microbiota of the PS150 group from both mouse models. Only genes with an estimated composition > 100 ppm of the metagenome were plotted. The top five coenriched genes are labeled with a red star, including histidine kinase, serine/alanine adding enzyme (*murM*), fructose-6-phosphate aldolase 1 (*fsaA*), aromatic-amino-acid transaminase (*tyrB*), and acetoin utilization protein (*acuB*). **(d)** Pathway mapping of the 410 coenriched genes from both models using the Kyoto Encyclopedia of Genes and Genomes (KEGG) database. Phosphotransferase (PTS) systems and the related carbohydrate metabolic pathways are colored red.

To predict the possible functional correlation of the remodeled microbiota, we conducted Phylogenetic Investigation of Communities by Reconstruction of Unobserved States (PICRUSt) analysis and interpreted the results using the Kyoto Encyclopedia of Genes and Genomes (KEGG) database. A total of nearly 4000 functional orthologs were present in the predicted metagenome, and a large portion of them were significantly enriched/reduced in the PS150-treated groups compared to the control groups. We filtered the predicted metagenome profile using the estimated fold difference in copy numbers (> twofold) and FDR-adjusted p-values (Q < 0.05) to identify significantly altered genes in the PS150-remodeled microbiota compared to the control group (Fig. 6a). We focused only on enriched genes for the predictively enhanced function of the remodeled microbiota. The results revealed a total of 472 and 745 enriched genes in the remodeled microbiota from the FNE and pentobarbital-induced sleep mouse models, respectively (Fig. 6b). Moreover, the estimated gene composition to the total metagenome (> 100 ppm) was used as a criterion to identify enriched genes with higher abundance. A total of 45 abundant genes were commonly enriched in the microbiota from both mouse models (Fig. 6c). The top 5 most enriched genes were histidine kinase, serine/alanine adding enzyme (*murM*), fructose-6-phosphate aldolase 1 (*fsaA*), aromatic-amino-acid transaminase (*tyrB*), and acetoin utilization protein (*acuB*). Furthermore, the 410 commonly enriched genes in both models (Fig. 6b) were related to multiple pathways, including amino acid metabolism, carbohydrate metabolism, gene regulation, chemical transportation, and biofilm formation (Fig. 6d).

## Discussion

*Lactobacillus fermentum* is considered a safe food supplement, and many strains have been shown to have health-improving properties^27^. *L. fermentum* PCC reduces severe gastrointestinal disorders and respiratory symptoms caused by weight-bearing training in athletes^28^. *L. fermentum* NS9 reduces symptoms of intestinal inflammation caused by antibiotics and reduces anxiety-like behavior and memory impairment^29^. Recently, the use of probiotics has been extended to improving sleep conditions. Heat-killed *Lactobacillus brevis* SBC8803 restored wakefulness and increased NREM sleep during the night in a stress-induced chronic sleep disorder mouse model^30^. *Lactobacillus casei* Shirota improved sleep quality under psychological stress^31^. Previously, we demonstrated a strain-specific effect caused by PS150 intervention on sleep latency using a chemically induced sleep mouse model^25^. In this study, we not only confirmed this effect in a spontaneous sleep model but also extended our understanding of the hypnotic effect of PS150 on sleep architecture. Furthermore, the effect of PS150 was compared to that of DIPH, a drug commonly used in over-the-counter sleep aids. Despite their low cost and availability, first-generation antihistamines, such as DIPH, have been reported to inhibit REM sleep, promote daytime sleep, and impair cognitive function^32^. In agreement with previous studies, we discovered that DIPH leads to REM sleep deprivation and fragmented NREM sleep in response to FNE (Figs. 2c and 3e). In particular, DIPH treatment led to a prolonged REM recovery compared to that in the control group, in accordance with the reported side effects (Fig. 4e). In contrast, none of the unwanted effects were induced by PS150, which indicates that it may be a better sleep aid than DIPH.

Excessive stress is considered to affect sleep efficiency and cause insomnia^33^. Many animal models, such as social defeat stress and novel environment exposure, are used to study the relationship between stress and sleep^34,35^. In addition, stressful events activate the HPA axis, which leads to the secretion of corticosteroids. Generally, hyperactivation of the HPA axis is considered to result in lighter and more fragmented sleep^36^. The bidirectional relationship between the HPA axis and the gut microbiota has been established ^37^. In this study, we found that administration of PS150 reduced serum corticosterone in the tail-handled group, suggesting a less active HPA axis. However, no significant HPA axis suppression was found under acute stress caused by cage changes, indicating that the hypnotic effect of PS150 may not result from reduced sensitivity to stress. Thus, we concluded that the hypnotic effect is achieved through pathways other than the HPA axis.

Elevated extracellular adenosine levels in the basal forebrain and hypothalamus promote slow-wave sleep and sleep duration^38,39^. Previous studies have indicated that adenosine inhibits the waking action of orexins and histaminergic neurons through A_1_R in the lateral hypothalamus and caudolateral hypothalamus^40–42^. Histaminergic neurons project from the tuberomammillary nucleus to the central nervous system and have been shown to promote wakefulness through Hist_1_R^43^. In addition, inosine produced by the gut microbiota can trigger A_2A_R signaling^44,45^. Although current studies are limited to the T-cell response, it still affects brain function because it efficiently passes through the blood–brain barrier^46,47^. In line with our previous study, we found that PS150 administration increased mRNA expression of A_1_R in the hypothalamus. However, the increased expression levels of A_1_R vanished under acute stress caused by cage changes. As there was no significant alteration in adenosine production (NR5e), adenosine signaling (A_1_R and A_2A_R), or histamine signaling (Hist_1_R), we concluded that PS150 may not function through either histamine- or adenosine-related pathways.

Studies have suggested a bidirectional relationship between the microbiota and host metabolism^48,49^. Furthermore, the diurnal rhythmicity of host gene expression was found to be regulated by the gut microbiota^50^. Evidence has shown that expression levels of host clock genes could be affected by several common microbial metabolites, including secondary bile acids, hydrogen sulfide, and short-chain fatty acids^51^. However, most studies have focused on the metabolic consequences and impact on microbiota caused by circadian dysregulation or sleep disruption^52^. Few studies have focused on the effect of altered microbiota on sleep quality. In this study, we demonstrated that the administration of PS150 leads to enrichment of the genera *Allobaculum*, *Bifidobacterium*, and *Coriobacteriaceae* UCG-002. Notably, *Bifidobacterium* is known to carry bile salt hydrolase genes (*bsh*) that convert host bile acids into secondary bile acids. In addition, *Allobaculum* belongs to Erysipelotrichaceae, which also generally carries *bsh*^51,53^. In contrast, only a portion of members of Coriobacteriaceae possess BSH activity^54^. Unfortunately, given the resolution limit of V3–V4 sequencing, we were unable to identify increased taxa at the species level. Therefore, we are unsure whether the increased Coriobacteriaceae produces secondary bile acids. The gut microbiota has been proven to modulate the host bile acid pool^55^. Furthermore, gut microbial *bsh* was found to regulate the transcription of key genes involved in lipid metabolism, cholesterol metabolism, gastrointestinal homeostasis, and circadian rhythm^56,57^. Taken together, we speculate that the remodeled microbiota may change the bile acid pool through BSH activity and that secondary bile acids could then affect the physiological activities of the host.

Prediction of the metagenome based on PICRUSt analysis revealed many enriched functional genes in the PS150-remodeled microbiota (Fig. 6b). In agreement with the significantly remodeled microbiota, the metagenomic profile of the PS150-treated groups was distinct from that of the control groups. One of the most enriched genes in the remodeled microbiota is *tyrB*, which encodes aromatic-amino-acid transaminase that catalyzes the reversible reaction to synthesize glutamate and aromatic oxo acids using 2-oxoglutarate and aromatic amino acids^58,59^. Enrichment of *tyrB* in the gut microbiota may potentially affect systemic aromatic amino acids, since a previous study demonstrated that oral administration of *Escherichia coli* expressing amino acid-degrading enzymes affects systemic phenylalanine availability in the host^60^. In addition, glutamate levels may also be affected by the enrichment of *tyrB*. This is supported by the fact that glutamate ABC transporter genes were also significantly increased in the remodeled microbiota (Fig. 6d). Glutamate receptors and transporters have been found to be universally expressed along the gastrointestinal tract and play an important role in the regulation of intestinal functions^61,62^. Moreover, supplementation with monosodium glutamate has been shown to regulate cognitive function, suggesting a possible effect of dietary glutamate on the brain^63^. However, since colonic glutamate makes nearly no contribution to the systemic glutamate concentration, the microbiota should only affect local glutamate levels in the colonic lumen^64^. Colonic luminal glutamate is a precursor of functional bacterial secondary metabolites, such as short-chain fatty acids^65^ and gamma-aminobutyric acid (GABA)^66^. As a result, the remodeled microbiota may affect the host through glutamate-dependent secondary metabolites. However, further studies are still required to determine the actual effect.

In addition to genes related to amino acid metabolism, genes related to carbohydrate metabolism, including galactose, fructose, mannose, amino sugars, and polysaccharide utilization pathways, were significantly enriched, as were their corresponding phosphotransferase (PTS) systems (Fig. 6d). Additionally, *fsaA*, which encodes fructose-6-phosphate aldolase and catalyzes the reversible reaction of synthesizing fructose 6-phosphate from dihydroxyacetone and D-glyceraldehyde 3-phosphate^67^, was one of the most enriched genes (Fig. 6c). These results suggest that the remodeled microbiota exhibit differential fermentation capability to carbon sources. Collectively, the predicted metagenome of the PS150-remodeled microbiota exhibited potential effects on the host metabolite pool and differential utilization of carbohydrates. To our knowledge, there is no direct evidence linking our functional prediction to the hypnotic effect. Additionally, further experimental evidence is required to elucidate the actual metabolic activity of the remodeled microbiota and its effect on host metabolism. It is still unclear how the remodeled microbiota may affect host behavior.

In conclusion, *L. fermentum* PS150 increases NREM sleep and reduces sleep latency. As a safe probiotic strain, PS150 effectively induces the hypnotic effect without the unwanted side effects of hypnotics, such as DIPH. Additionally, it can remodel the host microbiota, which may lead to changes in the host metabolite pool. Collectively, these data suggest that PS150 represents a promising hypnotic probiotic that may serve as a replacement for over-the-counter hypnotic drugs.

## Materials and methods

### Animals

All experimental mice used in this study were purchased from the National Laboratory Animal Center (Taipei, Taiwan) and housed in the animal center at National Yang-Ming University. All experiments were performed following relevant guidelines and regulations and were approved by the Institutional Animal Care and Use Committee of National Yang-Ming University (IACUC No. 1070311). Adult male C57BL/6J mice (6 weeks old) were used for all experiments. After the mice arrived at the Laboratory Animal Center of National Yang-Ming University, they were quarantined in a pathogen-free room for 2 weeks and checked for diseases. Mice were provided with water and chow ad libitum (Lab Diet Autoclavable Rodent Diet 5010; PMI Nutrition International, Brentwood, MO, USA). The room was soundproofed and kept at 22 ± 1 °C, 55%–65% humidity, and under a 12:12 h light/dark cycle. The light was turned on at 0900 h, which was defined as Zeitgeber time (ZT) 0, whereas 2100 h was defined as ZT 12.

### Preparation of *L. fermentum* PS150

PS150 used in the animal study was cultured in *Lactobacilli* Man Rogosa Sharpe (MRS) broth (BD Difco, MD, USA), anaerobically at 37 °C for 18 h, and then harvested by centrifugation at 4 °C and 10,000×*g* for 10 min. The supernatant was discarded, and the pellet was resuspended in MRS broth plus 12.5% glycerol to a final concentration of 5 × 10^9^ colony-forming units (CFU)/mL. The mixture was then aliquoted in tubes and stored at –20 °C until use. Each tube of the PS150 stock was then dissolved and washed twice using sterile PBS before use.

### Electrode implantation

Eight-week-old mice were intraperitoneally anesthetized using 50 mg/kg sodium pentobarbital and mounted on a stereotaxic apparatus before the surgery. Briefly, the dorsal surface of the skull was exposed, cleaned, and implanted with EEG and EMG electrodes for polysomnography. For the EEG electrode, three stainless-steel screws were driven bilaterally into the skull overlying the frontal lobe (+ 1 mm anterior to and –1 mm lateral to the bregma) and parietal cortex (1 mm posterior to and ± 1 mm lateral to the bregma) to fix the electrode device. A major electrode was inserted into the skull above the cortex (frontal lobe + 1 mm anterior to and + 1 mm lateral to bregma) as well as a reference electrode 2 mm caudal to the lambda. For the EMG electrode, two thin-strand stainless-steel microwires were inserted into the dorsal neck muscles to record the EMG. All electrodes were fixed onto the skull using dental cement, and the wounds were closed. After surgery, the mice were given antibiotics (chlortetracycline) and housed individually for 1 week to allow recovery. Wireless physiological sensor units were then placed on the head electrodes two days before the basal signal recording to allow adaptation. Polysomnographic recordings were performed, and subjects that failed to return normal signals were euthanized.

### Polysomnographic recording and analysis

Polysomnographic recordings were performed using a wireless physiological record sensor system (KY1C; K&Y Lab, Taiwan), as previously described^68^. Briefly, EEG and EMG signals were continuously recorded for 8 h (ZT 0 to ZT 8), and then the batteries were replaced. The next recording was performed for 12 h (ZT 12 to ZT 24) until the batteries ran out. Both EEG and EMG signals were amplified 1000-fold. For EEG, the signals were bandpass filtered over a frequency range of 0.16–53.05 Hz and digitalized using a 12-bit analog–digital converter with a 125 Hz sampling rate. For EMG, the signals were filtered at 0.72–112.88 Hz, and digitalized with a 250 Hz sampling rate. The digital signals were then wirelessly transmitted to a data recorder. EEG signals were then converted by fast Fourier transformation using a time window of 16 s with 50% overlap, and the intensities were integrated into delta (0.16–4 Hz), theta (6–9 Hz), sigma (10–14 Hz), and beta (14–32 Hz) powers^69^. EMG signals were converted by FFT using a time window of 2 s and were integrated according to designated frequencies (32–64 Hz). For determination of sleep–wake stages in each time epoch (8 s), three vigilance states were defined based on the product of sigma and theta powers (sigma*theta), delta power, and EMG power: wake (high EMG activity and low sigma*theta), NREM sleep (low EMG activity, high sigma*theta and high delta activity), and REM sleep (low EMG, high sigma*theta and low delta activity). As a final step, defined sleep–wake stages were visually examined and regulated if necessary.

### Cage change procedure

Mice were divided into six groups and orally administered 0.2 mL of PBS (TH-Veh, CC-Veh, TH-DIPH, or CC-DIPH) or 10^9^ CFU PS150 (TH-PS150 and CC-PS150) for 13 consecutive days. On day 14, mice were orally administered PBS (TH-Veh and CC-Veh), 4 mg/kg diphenhydramine hydrochloride (TH-DIPH and CC-DIPH), or PS150 (TH-PS150 and CC-PS150) immediately before the cage change procedure. The cage change procedure was performed at ZT 0 similarly as described^70,71^. For the cage change groups (CC-Veh, CC-DIPH, and CC-PS150), mice were transferred to a new cage with fresh bedding and food. For the tail-handled groups (TH-Veh, TH-DIPH, and TH-PS150), mice were removed by the tail and returned to their original cages to mimic transfer. Polysomnographic recordings were performed immediately after the cage change procedure. After completion of the recording on day 15, the cage change procedure was repeated, and mice were sacrificed. Tissue samples were stored at –80 °C until further use. This animal study was checked using SYRCLE’s risk of bias tool^72^ (Table S4) and was reported in compliance with the ARRIVE guidelines^73^.

### Corticosterone measurement

Blood samples were collected for estimation of corticosterone levels after treatment administration and repeated cage changes or tail handling on day 15. After centrifuging the blood at 3000 × *g* for 10 min, the serum was collected. We used a commercial corticosterone EIA kit (Cayman Chemical, Michigan, MI, USA) with diluted serum to analyze corticosterone concentrations following the manufacturer’s instructions.

### Quantitative real-time polymerase chain reaction (qRT-PCR) analysis

Total RNA was extracted from the brain tissue of mice using an RNeasy mini kit (Qiagen, Germantown, MD, USA) and was reverse transcribed to cDNA using a RevertAid First Strand cDNA Synthesis kit (Thermo Fisher, Waltham, MA, USA) for real-time PCR. cDNA samples were amplified using specific primers (Table S5) and KAPA SYBR FAST ABI Prism (KAPA Biosystems, Woburn, MA, USA) using the StepOnePlus Real-Time PCR System (Applied Biosystems, CA, USA). The target threshold cycle (Ct) was subtracted from the Ct for GAPDH to calculate ΔCt, and relative quantification analysis was performed using the 2^−ΔΔCT^ method^25^.

### Fecal sample collection, DNA extraction, and 16S rRNA library construction

For fecal sample collection, each mouse was placed in a clean cage until defecation. The feces were immediately collected into a 1.5 ml microtube containing 0.3 mL of RNAlater solution (Invitrogen) using sterilized forceps and placed on ice. The feces were then homogenized and washed twice with PBS. Fecal samples were then lysed with 0.2 mm glass beads using a bead beating (FastPrep) system, and metagenomic DNA was extracted using phenol–chloroform extraction. The quality of the DNA extracts was validated using nanodrops. For library construction, the prokaryotic rRNA V3–V4 hypervariable region was amplified using an adaptor containing a region-specific primer set. After amplification, index PCR was performed using amplified DNA to generate an indexed library for next-generation sequencing.

### V3–V4 16S rRNA sequencing and data analysis

Next generation sequencing and data processing were carried out by our service provider (Genewiz, South Plainfield, NJ, USA). For raw data collection, 2 × 300 bp paired-end (PE) sequencing was performed using a MiSeq v3 600 cycles kit on an Illumina MiSeq instrument following the manufacturer’s instructions. The adaptor sequences were removed using Cutadapt^74^ (v1.9.1), and the QIIME^75^ analysis package (v1.9.1) was used for 16S rRNA data analysis. PE reads were joined using overlapping sequences. The joined sequences were then trimmed to remove low-quality bases (Q_phred_ score < 20) from both the 5’ and 3’ ends. Raw reads with overlapping regions less than 20 bp or a total length shorter than 200 bp were discarded. SILVA 132 was used as the reference database, and chimera sequences were removed using UCHIME^76,77^. The resulting clean reads were subjected to operational taxonomic unit (OTU) clustering using VSEARCH^78^ (1.9.6) at a 97% threshold. For beta diversity analysis, beta distances between each sample were calculated based on Bray–Curtis and the UniFrac distance matrix. Intergroup differences were explored using either PCA or PCoA, and statistical significance was calculated by Adonis and Anosim analysis using the R package *vegan*^79,80^. Differential analysis of community composition was performed using the linear discriminant analysis effect size (LEfSE) method^81^. Metagenome prediction was performed using Phylogenetic Investigation of Communities by Reconstruction of Unobserved States (PICRUSt) analysis^82^. Differential analysis of the predictive metagenome was performed using Metastats^83^. The Kyoto Encyclopedia of Genes and Genomes (KEGG) database was used for interpretation and pathway mapping of the predictive metagenome^84^.

### Statistical analysis

Data were analyzed using GraphPad Prism software and are expressed as the mean ± standard error of the mean (SEM). We used one-way analysis of variance (ANOVA) with Tukey’s post hoc test for different groups. For comparison of effects from both the FNE and treatments, we conducted two-way ANOVA with Bonferroni correction. *p* < 0.05 was considered statistically significant.

## Supplementary Information

Supplementary Information.

## Data Availability

The V3–V4 16S rRNA sequencing data have been uploaded to NCBI BioProject (PRJNA689879).
