# Supplementary material for: Lactobacillus fermentum PS150 promotes non-rapid eye movement sleep in the first night effect of mice
Source: Sci Rep. 2021 Aug 11;11:16313. doi: 10.1038/s41598-021-95659-3 (PMC8357945; doi:10.1038/s41598-021-95659-3)
Supplement: Supplementary file 1 — Supplementary Information. [file 41598_2021_95659_MOESM1_ESM.pdf]

Lactobacillus fermentum PS150 promotes non-rapid eye movement sleep in the first night effect of mice

Alexander Lin[a,b‡], Ching-Ting Shih[b‡], Hsu-Feng Chu[c], Chieh-Wen Chen[d,e], Yu-Ting Cheng[d,e], Chien-Chen Wu[f], Cheryl C.H. Yang[d,e,g*], and Ying-Chieh Tsai[a*]

[a] Institute of Biochemistry and Molecular Biology, National Yang-Ming University, Taipei, Taiwan

[b] Chung Mei Biopharma Co., Ltd., Taichung, Taiwan

[c] Biomedical Industry Ph.D. Program, National Yang-Ming University, Taipei, Taiwan

[d] Institute of Brain Science, National Yang-Ming University, Taipei, Taiwan

[e] Sleep Research Center, National Yang-Ming University, Taipei, Taiwan

[f] Bened Biomedical Co., Ltd., Taipei, Taiwan

[g] Brain Research Center, National Yang-Ming University, Taipei, Taiwan

[‡] These authors contributed equally to this study and were listed according to alphabetical order.

[*] Correspondence:

Cheryl C.H. Yang, Ph.D., Institute of Brain Science, National Yang-Ming University, No. 155, Section 2, Linong Street, Beitou District, Taipei 11221, Taiwan

Tel: +886-2-2826-7058

E-mail: cchyang@ym.edu.tw

Ying-Chieh Tsai, Ph.D., Institute of Biochemistry and Molecular Biology, National Yang-Ming University, 155, Section 2, Linong Street, Beitou District, Taipei 11221, Taiwan

Tel: +886-2-2826-7125

E-mail: tsaiyc@ym.edu.tw

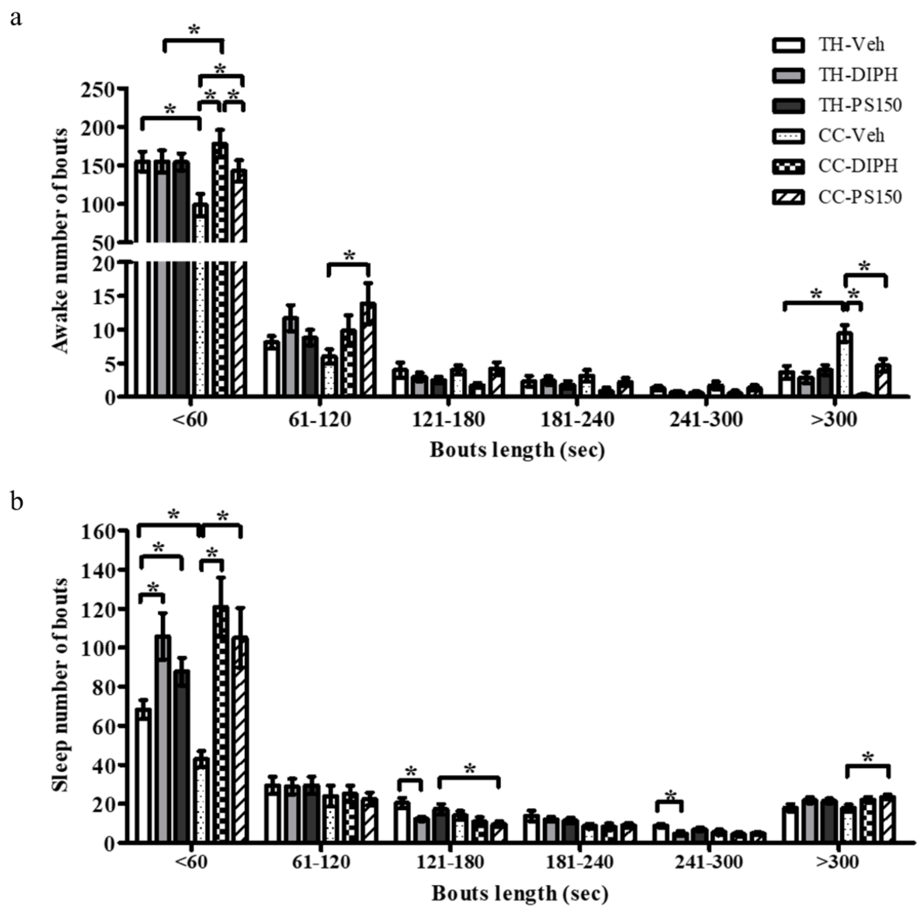

Figure S1. Distribution of wake and sleep bouts during the light phase. The length of bouts from each group in the light phase was calculated and categorized into six groups: <60, 61-120, 121-180, 181-240, 241-300, and >300 seconds. (a) Distribution of wake bouts. (b) Distribution of sleep bouts. Data are expressed as the mean ± SEM (n = 7–9) and were analyzed by two-way ANOVA with Bonferroni correction (* $p < 0.05$).

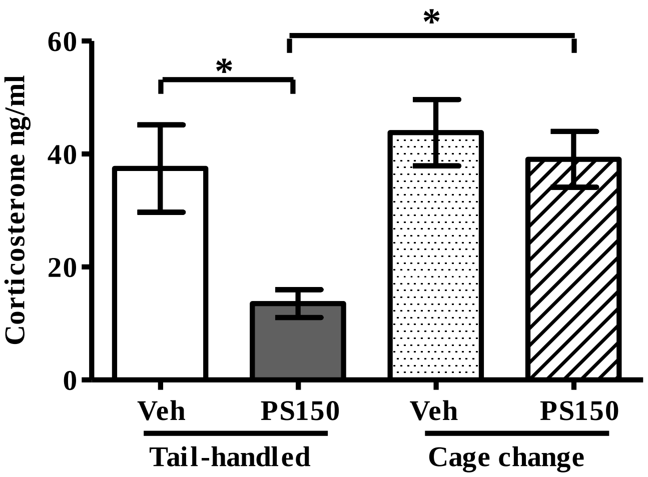

Figure S2. Changes in serum corticosterone levels after PS150 administration in mice. Mice were orally administered vehicle (phosphate-buffered saline) or PS150™ (10^9 CFU/day) on day 15, and the cage change procedure was repeated before sampling. Serum concentrations of corticosterone are expressed as the mean ± SEM ($n = 7$) and were analyzed by one-way ANOVA followed by Tukey's post hoc test. * $p < 0.05$, compared to each group.

a

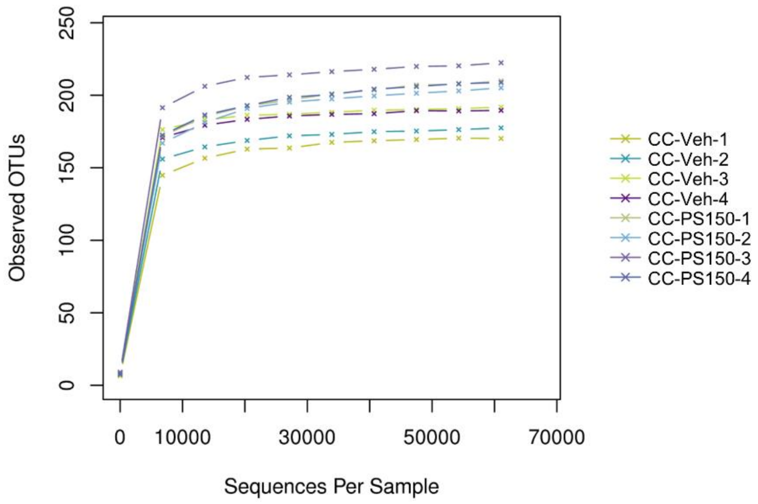

b

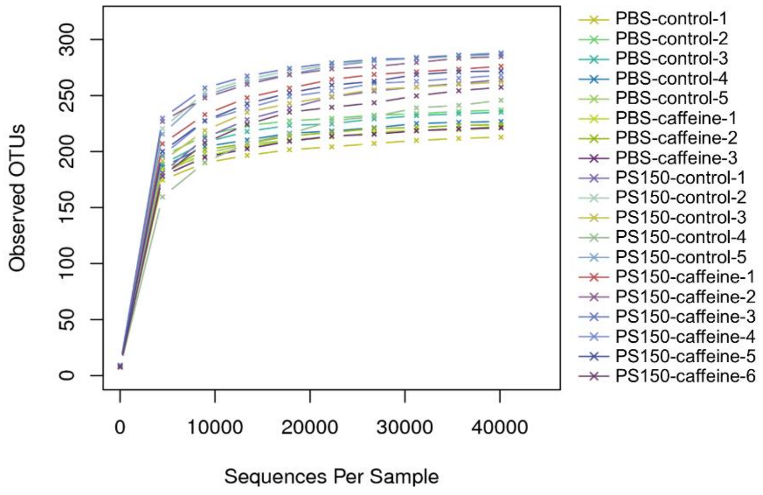

Figure S3. Rarefaction curve of the determined operational taxonomic unit (OTU) number. (a) Rarefaction curve of the determined OTU number from this study. (b) Rarefaction curve of the determined OTU number from the pentobarbital-induced sleep model[1].

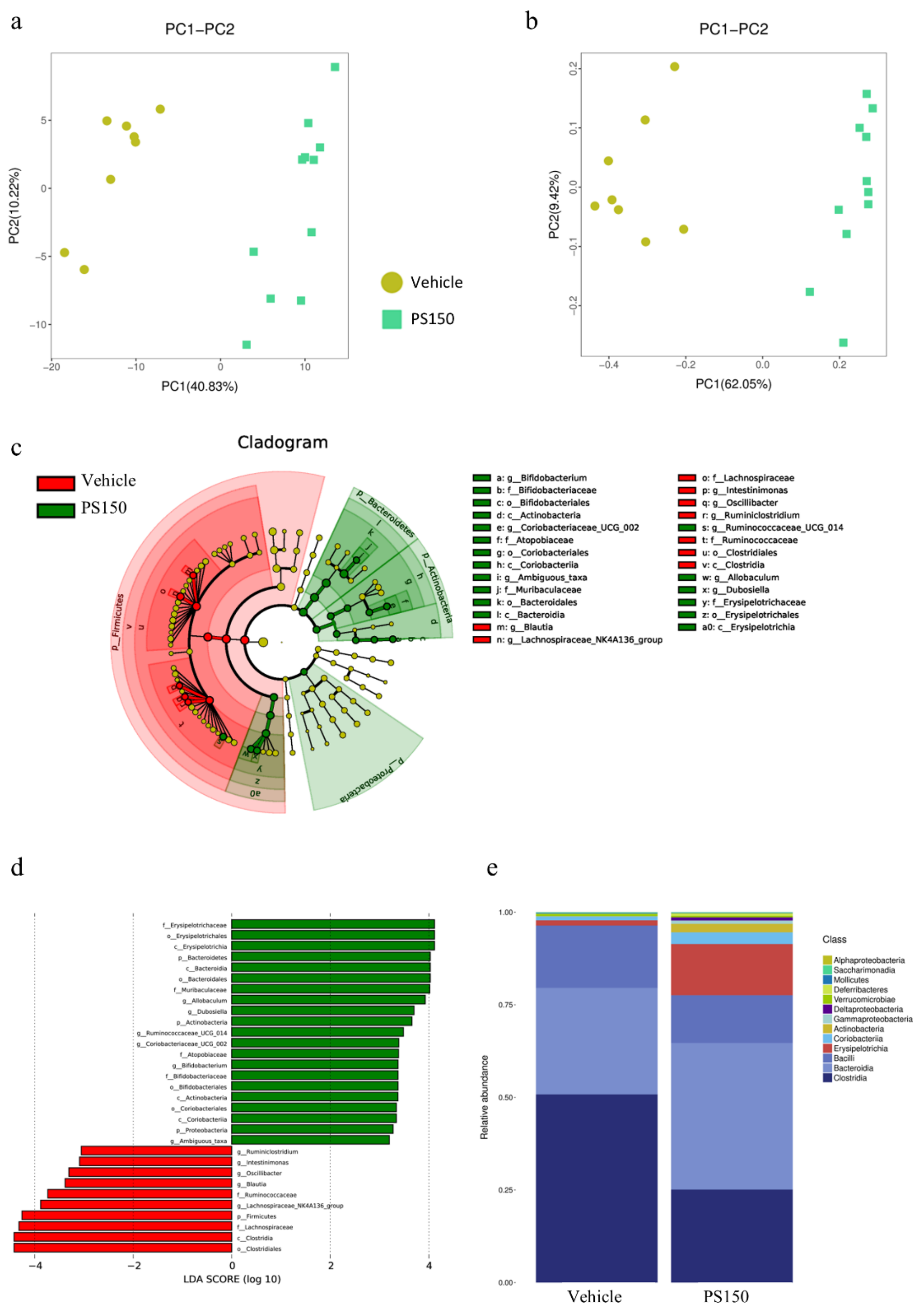

Figure S4. Differential microbiota composition of fecal specimens from naïve mice treated with PS150^{TM} for 4 weeks. (a) Principal component analysis (PCA) and (b) principal coordinates analysis (PCoA) plot of fecal microbiota. (c) Cladogram from linear discriminant analysis effect size (LEfSE) analysis. Enriched taxa in either the vehicle control or PS150 group are colored red and green, respectively. (d) LDA score from LEfSE analysis. (e) Stacked plot of relative microbial distribution at the class level.

Table S1. Quantitative real-time polymerase chain reaction (qRT-PCR) analysis of mRNA expression in the basal forebrain and hypothalamus.

	Basal forebrain				Hypothalamus			
	Tail-handled		Cage change		Tail-handled		Cage change	
	Veh	PS150	Veh	PS150	Veh	PS150	Veh	PS150
A_1R	1.00± 0.11	1.08± 0.30	1.00± 0.22	1.11± 0.24	1.00± 0.14	1.37± 0.40*	1.07± 0.33	1.31± 0.23
$A_{2A}R$	1.00± 0.27	0.97± 0.21	0.77± 0.31	0.85± 0.30	1.00± 0.41	1.20± 0.83	1.18± 0.65	1.46± 0.91
NT5e	1.00± 0.22	1.13± 0.29	0.86± 0.18	0.99± 0.21	1.00± 0.21	1.04± 0.30	0.98± 0.33	1.08± 0.29
$Hist_1R$	1.00± 0.16	1.09± 0.33	0.84± 0.30	0.97± 0.26	1.00± 0.35	1.35± 0.48	1.07± 0.63	1.34± 0.39

Gene expression was analyzed in the basal forebrain and hypothalamus of mice. Data are expressed as the mean ± SEM (n = 7–8) and were analyzed by one-way ANOVA with Tukey's post hoc test; * $p < 0.05$, compared to the Veh group; A_1R, adenosine A_1 receptor; $A_{2A}R$, adenosine A_{2A} receptor; $Hist_1R$, histamine $_1$ receptor; NT5e, ecto-5′-nucleotidase.

Table S2. Summary of data quality.

Model	Group	PE reads*	Q30(%)*	Clean reads#	Average# length (bp)	GC (%)#	Source
The first-night effect	PS150 (n=4)	106715±14156	82.2±0.6	85595±12292	458±2	54.7±0.2	This study
	PBS (n=4)	112168±9090	80.8±1.1	92267±7088	458±3	54.7±0.2	
Pentobarbital induced sleep	PS150 (n=11)	97218±16860	81.3±0.7	81830±12788	459±2	54.5±0.3	[1]
	Control (n=8)	99620±16904	82.5±0.7	77948±14539	455±4	54.4±0.3	

The mean value and standard deviation of the jointed raw data (*) and trimmed data (#) are listed.

Table S3. Top altered genera discovered using Metastats analysis.

Model	Genus	PS150^{TM}* (%)	Control* (%)	P-value	Q value	Source
Pentobarbital induced sleep	Lachnospiraceae NK4A136 group	3.73±1.00	11.33±1.56	<0.001	<0.05	[1]
	Allobaculum	7.90±1.15	ND	<0.001	<0.05	
	Dubosiella	4.72±2.00	ND	<0.001	<0.05	
	Unclassified Ruminococcaceae	0.65±0.13	3.4±0.38	<0.001	<0.05	
	Coriobacteriaceae UCG-002	2.30±0.23	ND	<0.001	<0.05	
	Bifidobacterium	2.27±0.32	0.03±0.03	<0.001	<0.05	
	Oscillibacter	0.30±0.09	2.15±0.21	<0.001	<0.05	
	Ruminiclostridium	0.13±0.04	1.17±0.16	<0.001	<0.05	
	Intestinimonas	0.08±0.03	1.22±0.24	<0.001	<0.05	
The first-night effect	*Enterorhabdus*	0.08±0.01	1.22±0.16	<0.001	<0.001	This study
	Allobaculum	8.94±1.39	ND	0.002	0.28	
	Marvinbryantia	0.01±0.00	1.13±0.18	0.003	0.37	
	Desulfovibrio	1.18±0.24	ND	0.006	0.51	
	Ruminococcaceae UCG-014	3.47±0.51	0.78±0.29	0.007	0.52	
	Coriobacteriaceae UCG-002	2.33±0.54	ND	0.009	0.53	
	Faecalibaculum	1.28±0.43	ND	0.024	1	
	Bifidobacterium	3.78±1.30	ND	0.028	1	
	Unclassified Muribaculaceae	44.66±2.03	56.27±3.57	0.029	1	
	Unclassified Ruminococcaceae	0.44±0.11	1.12±0.22	0.032	1	

Relative abundance of microbiota composition at the genus level (*). Taxa with relative amounts less than 0.01% were labeled ND.

Table S4. Risk of bias in this study was evaluated using SYRCLE’s risk of bias tool.

Item	Type	Domain	Risk	Descriptions
1	Selection	Sequence generation	Unclear	Animals were randomly assigned to each group manually.
2	Selection	Baseline characteristics	Low	Baselines were confirmed insignificant between groups before treatment.
3	Selection	Allocation concealment	High	The grouping was assigned by investigators.
4	Performance	Random housing	Low	Mice were individually housed in cages and randomly placed within the animal room.
5	Performance	Blinding	High	The investigators were not blinded in this study.
6	Detection	Random outcome assessment	High	Animals were picked according to designated sequence during outcome assessment.
7	Detection	Blinding	High	The investigators were not blinded in this study.
8	Attrition	Incomplete outcome data	Low	Data were only removed due to technical failure.
9	Reporting	Selective outcome reporting	Low	All outcomes were reported.
10	Other	Other sources of bias	Low	The sponsor had no role in the analysis and interpretation of data.

Table S5. Primers used in this study.

	Sequence (5’→3’)	Size (bp)	Accession number
A_1R-F	AGAACCACCTCCACCCTTCT	227	XM_006529079.2
A_1R-R	TACTCTGGGTGGTGGTCACA		
A_{2A}R-F	AACCTGCAGAACGTCAC	245	XM_006513093.3
A_{2A}R-R	GTCACCAAGCCATTGTACCG		
$Hist_1$R-F	GACAAGATGTGTGAGGGGAA	285	XM_006505616.3
$Hist_1$R-R	CCATAGAGAGCCAAAAGAGG		
NT5e-F	TTACTAAAGCATGACTCTGGTGATCAA	84	NM_011851.4
NT5e-R	AACGGCTGGGTAAACTACTTTCATT		
GAPDH-F	CAATGTGTCCGTCGTGGATCT	208	XM_017321385.1
GAPDH-R	GTCCTCAGTGTAGCCCAAGATG		

A_1R, adenosine A_1 receptor; A_{2A}R, adenosine A_{2A} receptor; $Hist_1$R, histamine $_1$ receptor; NT5e, 5′ nucleotidase-ecto (the enzyme converts adenosine monophosphate to adenosine); GAPDH, glyceraldehyde 3-phosphate dehydrogenase

SUPPLEMENTARY REFERENCES

1 Lin, A. *et al.* Hypnotic Effects of *Lactobacillus fermentum* PS150^{TM} on Pentobarbital-Induced Sleep in Mice. *Nutrients* **11**, doi:10.3390/nu11102409 (2019).
